# Inferring condition-specific targets of human TF-TF complexes using ChIP-seq data

**DOI:** 10.1186/s12864-016-3450-3

**Published:** 2017-01-10

**Authors:** Chia-Chun Yang, Min-Hsuan Chen, Sheng-Yi Lin, Erik H. Andrews, Chao Cheng, Chun-Chi Liu, Jeremy J.W. Chen

**Affiliations:** 1 Institute of Molecular Biology, National Chung Hsing University, Taichung, Taiwan; 2 Institute of Genomics and Bioinformatics, National Chung Hsing University, Taichung, Taiwan; 3 Institute of Biomedical Sciences, National Chung Hsing University, No. 250, Kuo-Kuang Rd., 40227 Taichung, Taiwan; 4 Agricultural Biotechnology Centre, National Chung Hsing University, Taichung, Taiwan; 5 Department of Genetics, Geisel School of Medicine at Dartmouth, 03755 Hanover, NH USA; 6 Institute for Quantitative Biomedical Sciences, Geisel School of Medicine at Dartmouth, 03766 Lebanon, NH USA

**Keywords:** Transcription factor, TF-TF complexes, Condition-specific target, ChIP-seq, Database

## Abstract

**Background:**

Transcription factors (TFs) often interact with one another to form TF complexes that bind DNA and regulate gene expression. Many databases are created to describe known TF complexes identified by either mammalian two-hybrid experiments or data mining. Lately, a wealth of ChIP-seq data on human TFs under different experiment conditions are available, making it possible to investigate condition-specific (cell type and/or physiologic state) TF complexes and their target genes.

**Results:**

Here, we developed a systematic pipeline to infer Condition-Specific Targets of human TF-TF complexes (called the CST pipeline) by integrating ChIP-seq data and TF motifs. In total, we predicted 2,392 TF complexes and 13,504 high-confidence or 127,994 low-confidence regulatory interactions amongst TF complexes and their target genes. We validated our predictions by (i) comparing predicted TF complexes to external TF complex databases, (ii) validating selected target genes of TF complexes using ChIP-qPCR and RT-PCR experiments, and (iii) analysing target genes of select TF complexes using gene ontology enrichment to demonstrate the accuracy of our work. Finally, the predicted results above were integrated and employed to construct a CST database.

**Conclusions:**

We built up a methodology to construct the CST database, which contributes to the analysis of transcriptional regulation and the identification of novel TF-TF complex formation in a certain condition. This database also allows users to visualize condition-specific TF regulatory networks through a user-friendly web interface.

**Electronic supplementary material:**

The online version of this article (doi:10.1186/s12864-016-3450-3) contains supplementary material, which is available to authorized users.

## Background

Transcription factors (TFs) interact with one another and with their co-factors to form TF complexes, with constituents that vary in different cell types or under different cellular conditions. These TF complexes regulate different sets of target genes to determine cellular state [1]. Given the high variability of TF complex composition, it is critical to examine TF complexes and their target genes in a condition-specific manner to accurately reveal their regulatory activities.

TF-TF interactions can be experimentally identified using electrophoretic mobility shift assays (EMSAs), X-ray crystallography, immunoprecipitation, yeast two-hybrid systems, mammalian two-hybrid systems and luciferase assays. Because of technical limitations, most human TF-TF interactions represent potentials of physical binding rather than physiological interactions under specific conditions. For example, Ravasi et al. developed a database of physical TF-TF interactions using a mammalian two-hybrid system in hamster cells [2], in which approximately 1,600 TF-TF interactions were identified among human and mouse TFs. However, these data merely indicated the potential interactions amid the pertinent TF pairs in the experimental model. These data did not reflect the condition-specific target genes of TF-TF complexes, which are essential for understanding their regulatory mechanisms.

Chromatin immunoprecipitation followed by DNA microarray or high-throughput sequencing (ChIP-chip/ChIP-seq) techniques are powerful for identifying TF binding sites. These approaches discover binding “peaks”, i.e. regions of chromatin and the corresponding sequences enriched for TFs. Consequently, condition-specific TF peaks can be identified by altering cellular conditions, which further reveal motifs recognized by DNA-binding TFs or their co-regulatory counterparts. For example, the CENTDIST web server identifies co-regulatory TFs in complexes by investigating TF motifs enriched in the ChIP-seq peaks for a TF [3]. In addition, the spacing of TF-pair binding motifs is often inflexible [4], allowing the SpaMo algorithm to identify TF-TF pairs by interrogating motif spacings [5]. However, in light of many binding peaks having been shown to be non-functional [6, 7], such methods may not be informative for identifying functional binding sites.

Thanks to a comprehensive TF motif database, CST is the first pipeline that uses data from a single ChIP-seq experiment to predict both TF partners and their target genes. Chen et al. predicted TF complexes and their target genes using yeast TF ChIP-chip data [8], but their method required paired ChIP-chip data: one assay to determine the binding sites of a primary TF and the other the binding sites of a partner TF. Therefore, we believe CST will lower the cost of using ChIP-seq for these purposes and be valuable to the community.

CST uses ChIP-seq data after immunoprecipitation of the primary TF along with a database containing known TF binding sequence motifs to identify partner TFs. Finally, we integrated the predicted results and constructed a database called DBCST. DBCST allows users to upload their own ChIP-seq data and analyse them for TF complexes and their regulatory targets. DBCST is freely available at http://syslab3.nchu.edu.tw/DBCST.

## Results

### Prediction of TF complexes and their target genes

**Fig. 1:**
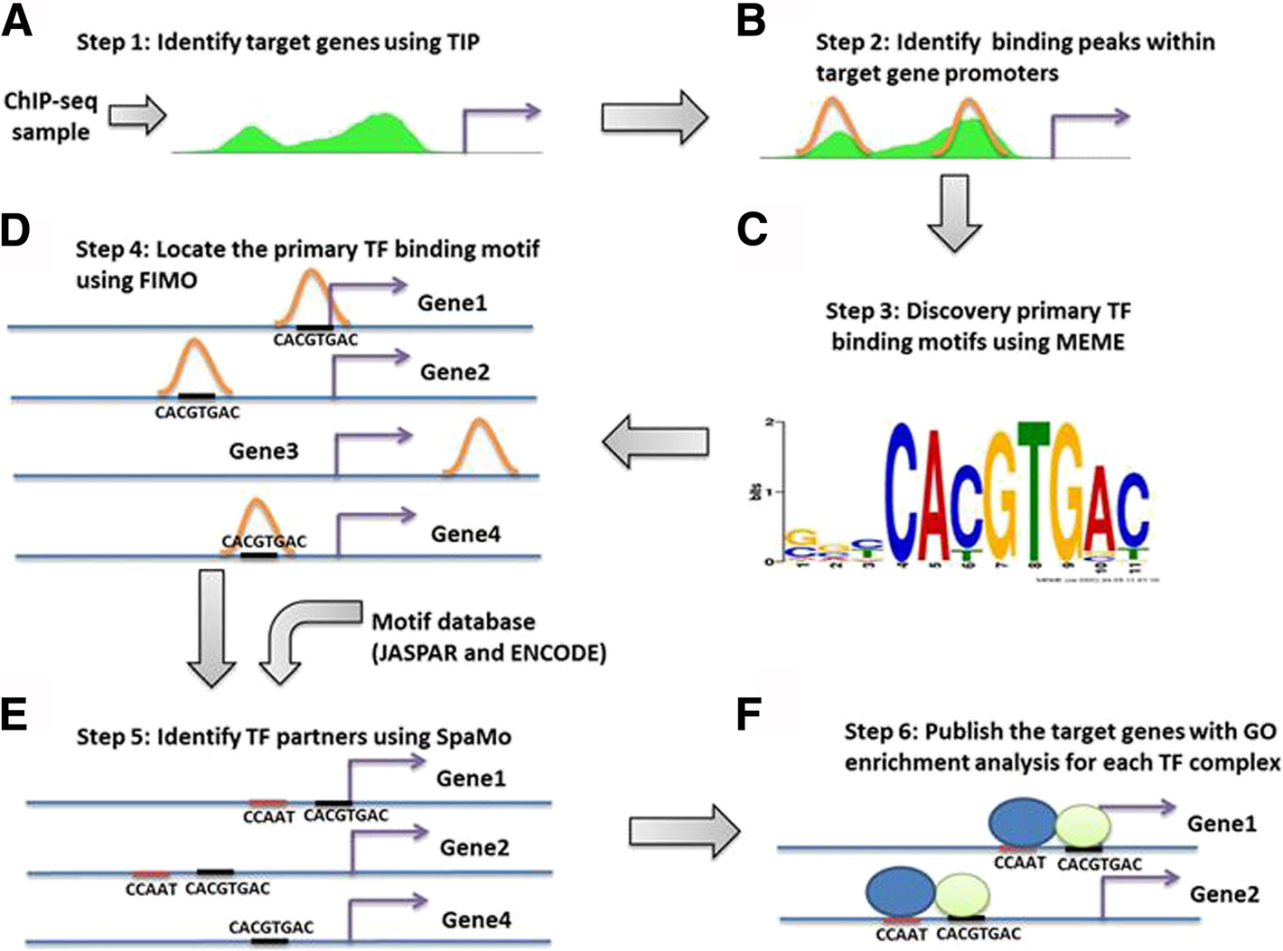
Overview of the CST pipeline. **a** Given a ChIP-seq sample, primary TF target genes are identified using the TIP algorithm. **b** For motif discovery, the binding peaks on target gene promoters are first identified using the narrow peaks located in the putative promoters of the TIP-predicted target genes. **c** For binding motif discovery, the binding peaks on the target genes are selected, and MEME is used to discover primary TF binding motifs. **d** FIMO is used to locate the primary TF binding motifs in the binding peaks of the primary TF target genes. **e** Using the binding motifs generated from MEME for all TFs and adding in motifs from the JASPAR database, SpaMo is used to search for binding motifs of potential partner TFs and to analyse their statistical significances based on their spacings. **f** The resulting predicted TF complexes and their target genes are reported with GO enrichment results. Target genes are stratified into high- and low-confidence groups based on the SpaMo-calculated statistical significance of their TF complex binding motif spacing

Using high-confidence criteria (see Methods and Fig. 1); our pipeline identified 13,504 relationships between 2,392 predicted TF complexes and 3,272 predicted target genes. By contrast, when using low-confidence criteria (see Methods and Fig. 1), we identified 127,994 relationships. In addition, the correlation between gene expression and TF binding was highly significant (*P* = 2.2× 10^−16^, see Additional file 1 Supplementary Methods) and the likelihood of a TF complex near transcriptionally active genes showed that the TF complexes are most likely located -1kbp to 0.5kbp around TSS (Fig. S1). The numbers of ChIP-seq datasets for each cell line used in our database are provided in Additional file 1: Table S1. The high-confidence and low-confidence target genes of the predicted USF2-NFYA complex using the ChIP-seq data for USF2 in K562 cells are partially listed in Fig. S2. Brief instructions for users and a detailed tutorial of DBCST can be found in the Additional file 1 Supplementary Information and on the web page, respectively.

### Validation of CST-predicted TF complexes by comparison to other databases

**Fig. 2:**
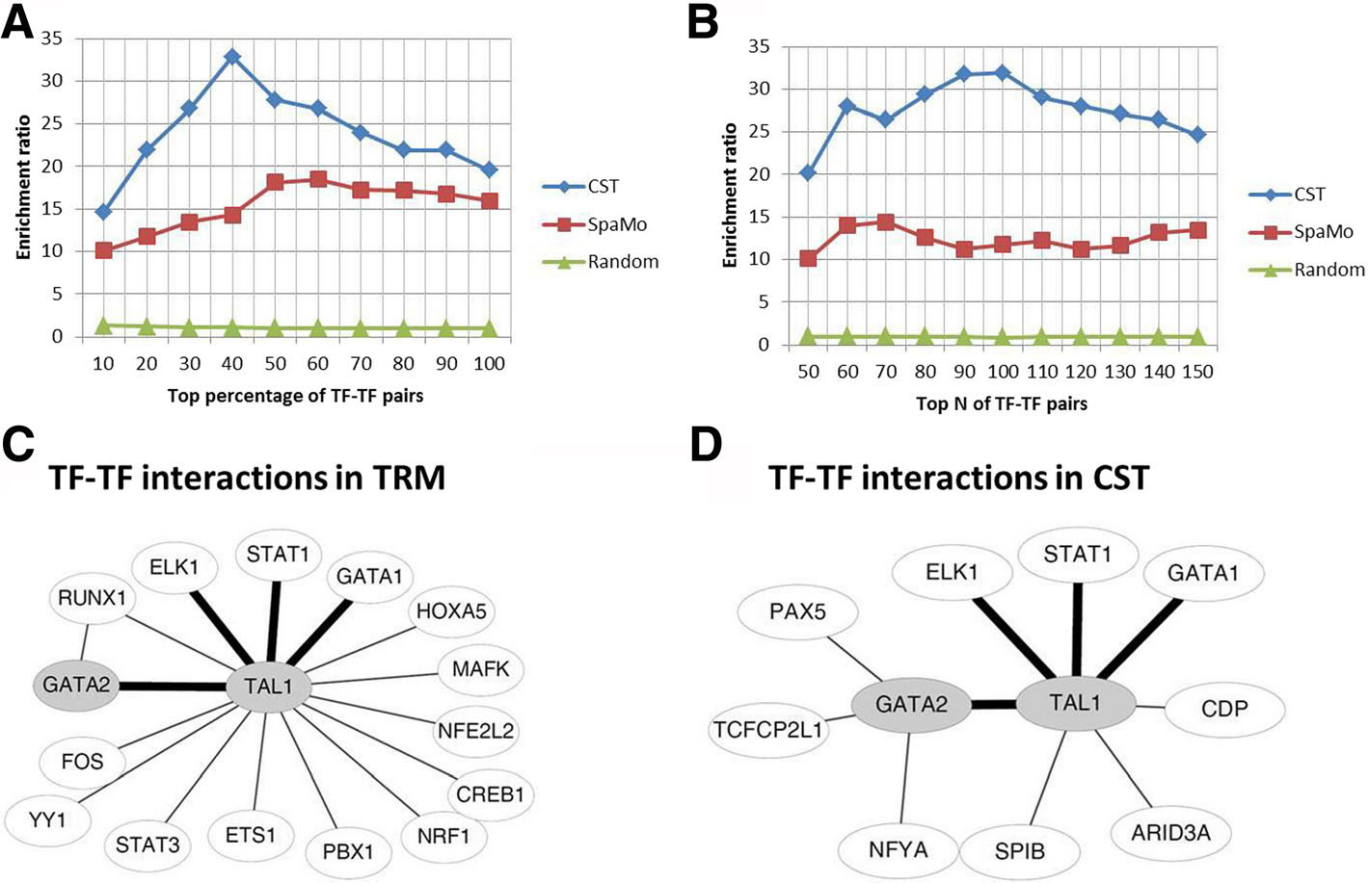
Comparison and validation of CST-predicted TF complexes. In (**a**) and (**b**), we compared the presence of CST-predicted TF complexes relative to SpaMo-predicted TF complexes in an external, experimentally derived database of TF complexes to demonstrate the performance of CST. **a** The x-axis represents the TF complexes ordered by their SpaMo-calculated p-values (from most to least significant), and the y-axis represents the enrichment ratio. The best enrichment ratios of CST and SpaMo were approximately 32 and 18, respectively. CST has greater enrichment than SpaMo across all p-values. The enrichment ratio was calculated as the ratio of predicted TF complexes in the database relative to the number of 1000 randomly generated TF complexes in the database. **b** Similar to (**a**), the top N of TF complexes calculated by p-values are used. The best enrichment ratios of CST and SpaMo were approximately 32 and 14, respectively. CST demonstrated greater enrichment than SpaMo across the entire N range. In (**c**) and (**d**), we validated the condition-specific TF-TF interactions using TRMs to demonstrate the condition-specific accuracy. The nodes are TFs, and the edges indicate interactions. GATA2 and TAL1 (grey colour) are present in both TRM and ENCODE ChIP-seq data. Combined GATA2 and TAL1 TRMs in HSCs contained 16 TF-TF interactions (**c**), whereas 10 predicted TF-TF interactions were identified in CST using GATA2 and TAL1 ChIP-seq data in K562 cells (**d**). The bold edges indicate TF-TF interactions common between TRMs and CST. Four significant TF complexes between TRMs and CST are indicated with bold edges (*P* = 3*10^−4^; Fisher’s exact test), suggesting the consistency of TRM and CST

To evaluate CST, we examined the presence of predicted condition-specific TF complexes in two external databases. The first database was to demonstrate the performance of CST (Fig. 2a and b), whereas the second was to investigate the accuracy in a condition-specific circumstance (Fig. 2c and d).

For the first validation, we compared the degree of enrichment for the CST-predicted TF complexes present in an empirically determined TF complex database (see Methods) against that of TF complexes created randomly among potential TF pairs in the CST pipeline (i.e. a background). We also included TF complexes predicted by SpaMo [5] for a fair comparison (Fig. 2a and b). After ordering the TF complexes by *p*-values in an ascending manner and calculating enrichment ratios, we discovered that TF complexes identified by CST were highly enriched compared with those by SpaMo. The peak enrichment for CST was approximately 32 (at the 40% confidence decile), whereas that for SpaMo was approximately 18 (at the 60% decile). These results indicated that Target Identification from Profiles (TIP) method [9] together with SpaMo, equivalent to CST, significantly improved the prediction of TF complexes over the use of SpaMo alone. Similar results are suggested in Fig. 2b, in which the top N of TF complexes are selected.

For the second validation, we compared the CST-predicted TF complexes to the TF-specific transcriptional regulatory modules (TRMs) in haematopoietic stem cells (HSCs) proposed by Diez et al. (see Methods) [10]. Briefly, Diez et al. used ChIP-seq to identify condition-specific binding sites. After scanning enriched motifs in these binding sites and integrating protein-protein interaction data, the authors discovered condition-specific TRMs for each immunoprecipitated TF. Although there were 9 TRMs in HSCs (ERG, FLI1, GATA2, GFI1B, LMO2, MEIS1, SFPI1, RUNX1 and TAL1), two TRMs were observed in CST (including GATA2 and TAL1 in K562 cells). Four significant TF complexes were observed in both TRM and CST (3 in the TAL1 and 1 in the GATA2 datasets; *P* = 3*10^−4^; Fisher’s exact test) after further comparisons for the TRM-predicted TF complexes (Fig. 2c) and for the CST-predicted TF complexes (Fig. 2d). In order to compare CST and SpaMo predictions, Additional file 1: Figure S3 shows SpaMo-predicted TF complexes from GATA2 and TAL1 ChIP-seq data in K562 cells. The result of CST is more significant than the SpaMo prediction (P = 0.02; Fisher’s exact test). Notably, the predicted motif spacings of TAL1-STAT1 and TAL1-GATA1 interactions in CST are 85 and 23 bps, respectively (Additional file 1: Table S2). According to a previous study claiming that a TF-TF interaction is likely indirect if the spacing of the interaction exceeds 30 bps [11], we speculated that interactions between TAL1 and STAT1 are indirect, whereas between TAL1 and GATA1 are direct. This result is consistent with the TRM database, in which TAL1 indirectly interacts with STAT1 by the WDR5 bridge protein, whereas TAL1 directly interacts with GATA1.

### Validation of CST-predicted target genes using ChIP-qPCR and RT-PCR

Using USF2 ChIP-seq data in K562 cells, our pipeline predicted that USF2 and NFYA form a TF complex that possesses a significant motif spacing of 9 bps and binds to five genomic locations, regulating eight target genes with high confidence (Table 1). USF2 is a basic helix-loop-helix leucine zipper protein recognizing the E-box (CACGTG) DNA-binding motif, whereas NF-Y is a trimeric TF consisting of two histone-like subunits (NFYB and NFYC) and a CCAAT binding subunit (NFYA). Using EMSA, Zhu et al. reported that USF2 and NFYA form a TF complex at the HoxB4 promoter in K562 cells [12], which supports our prediction that USF2 and NFYA together form a TF complex in K562 cells.

**Table 1:** Eight high-confidence target genes of the USF2-NFYA complex derived from K562 USF2 ENCODE ChIP-seq data

Location of motif pairs^a^	Target gene^b^	Motif spacings^c^
Chr14: 20923275-20923304	OSGEP, APEX1	9 bps
Chr7: 108210264-108210293	THAP5, DNAJB9	9bps
Chr4: 99850329-99850358	EIF4E	9bps
Chr16: 4897410-4897439	GLYR1, UBN1	9bps
Chr12: 104359548-104359577	TDG	9bps

^a^The location of the predicted USF2 and NFYA motif pair from K562 USF2 ChIP-seq data and the motif database

^b^The target genes for which the motif pairs occur in their putative promoters and are TIP-derived target genes of USF2 (the primary TF)

^c^The spacing of the USF2-NFYA motif pairs on the putative promoters of the target genes

**Fig. 3:**
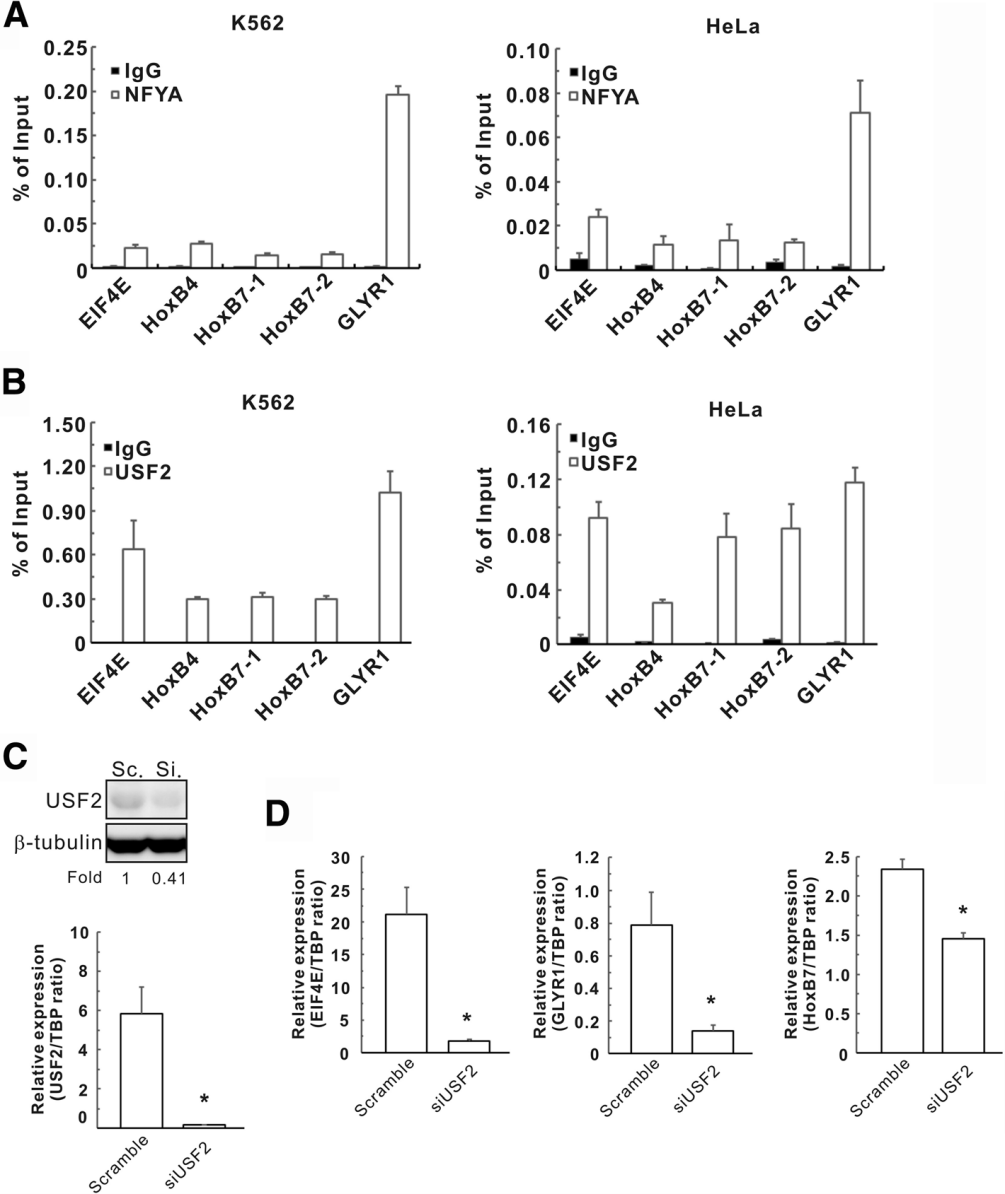
Validation of predicted targets of the USF2-NFYA complex using ChIP-qPCR and RT-PCR. **a** ChIP-qPCR with a NFYA pull-down and qPCR amplification against CST NFYA-USF2-predicted target genes. The genomic DNA from K562 cells (left panel) and HeLa cells (right panel) that immunoprecipitated with NFYA and nonspecific IgG antibodies was used for qPCR to assess the fold enrichment of the respective gene promoters in NFYA-IP DNA over IgG-IP for each gene. The fold enrichments were the averages of three independent experiments and the data were presented as the means ± standard errors. HoxB4 was used as a positive control (see Methods). **b** Same as (**a**) with a USF2 pull-down. **c** The expression level of USF2 in HeLa cells with USF2 silencing by siRNA. Upper panel: Western blot; β-tubulin: internal control. Lower panel: real-time RT-PCR; TBP: internal control. **d** The expression levels of three downstream genes of USF2 in HeLa cells with USF2 silencing, as determined by real-time RT-PCR. *P < 0.05, compared with scramble control

To experimentally validate the interactions between the predicted USF2-NFYA TF complex and its targets, we used ChIP-qPCR in K562 and HeLa cells for *in vitro* validation. For qPCR amplification targets, we selected the promoters of high-confidence target genes (EIF4E and GLYR1), a low-confidence target gene (HoxB7) and HoxB4 (a positive control according to Zhu et al.). To ensure PCR accuracy, we designed two primer sets for HoxB7 (see Additional file 1: Table S3). Relative to IgG-IP normalization, the qPCR fold enrichment of all targets was large and highly significant for both NFYA (Fig. 3a) and USF2 (Fig. 3b) in both K562 and HeLa cell lines. In addition, the regular PCR amplification from USF2-IP and NFYA-IP DNA also demonstrated the interaction between the USF2-NFYA complex and the promoter of the target genes (Additional file 1: Fig. S4). Although these ChIPs against USF2 and NFYA were independent of one another, the results supported the conclusion that both TFs bind to the same target sequences on target genes predicted in CST.

To experimentally validate that these targets were activated by USF2-NFYA complex in HeLa cells, we interfered with the complex formation by silencing USF2 (Fig. 3c) and then investigated the expression levels of three downstream genes with real-time RT-PCR (Fig. 3d). The data clearly demonstrated that the expression levels of predicted target genes (EIF4E, GLYR1 and HoxB7) of the USF2-NFYA complex were reduced while USF2 was silenced.

### Clustering target genes for each TF complex separately results indifferent GO enrichment results

Changes in the interacting partner (s) of a given TF often result in alterations in target genes to elicit different biological functions. To examine these phenomena, we performed gene ontology (GO) enrichment analysis of target genes for given TF complexes to determine how they varied with TF complex composition. Using USF2 ChIP-seq data in K562 cells, CST predicted that USF2 and IRF1 form a TF complex. Among the top 10 GO enrichment results for the USF2-IRF1 complex targets (Table 2, upper panel), the second, third and tenth GO terms are related to iron transport. Previous studies reported that USF2 and IRF1 co-regulate β_2_-microglobulin, which can regulate iron metabolism and transport [13, 14]. This result is completely different from the GO enrichment results of the predicted USF2-NFYA TF complex targets, which are related to DNA catabolic processes and activity (Table 2, lower panel). CST fully distinguishes the functionality of the USF2-IRF1 complex from that of USF2-NFYA, implying that the USF2-IRF1 and USF2-NFYA TF complexes recruit different downstream target genes to determine phenotypes in K562 cells.

**Table 2:** A partial list of the GO analysis results of target genes predicted by two different putative complexes using the K562 USF2 ChIP-seq data

Rank	Enrichment GO term	*p*-value
Predicted USF2-IRF1 complex^a^		
1	GO:0032870 cellular response to hormone stimulus	2.15E-07
2	GO:0033572 transferrin transport	7.16E-07
3	GO:0015682 ferric iron transport	7.16E-07
4	GO:0071495 cellular response to endogenous stimulus	1.20E-06
5	GO:0071375 cellular response to peptide hormone stimulus	1.55E-06
6	GO:0015031 protein transport	2.94E-06
7	GO:0044437 vacuolar part	3.21E-06
8	GO:0005654 nucleoplasm	3.82E-06
9	GO:0043434 response to peptide hormone stimulus	7.11E-06
10	GO:0006826 iron ion transport	7.92E-06
Predicted USF2-NFYA complex^b^		
1	GO:0004536 deoxyribonuclease activity	1.53E-04
2	GO:0016798 hydrolase activity, acting on glycosyl bonds	2.58E-04
3	GO:0019104 DNA N-glycosylase activity	3.79E-04
4	GO:0006886 intracellular protein transport	5.00E-04
5	GO:0044419 interspecies interaction between organisms	5.43E-04
6	GO:0006308 DNA catabolic process	6.97E-04
7	GO:0044265 cellular macromolecule catabolic process	7.91E-04
8	GO:0016799 hydrolase activity, hydrolysing N-glycosyl compounds	1.16E-03
9	GO:0060674 placenta blood vessel development	1.29E-03
10	GO:0032507 maintenance of protein location in cell	1.42E-03

^a^GO enrichment results for targets of the predicted USF2-IRF1 complex. The second, third and tenth GO terms are related to iron transport. A previous study reported that USF2 and IRF1 co-regulate β2-microglobulin, which regulates iron metabolism and transport

^b^GO enrichment results for targets of the predicted USF2-NFYA complex. The top ten GO term results are associated with DNA catabolism and clearly differed from the results of the predicted USF2-IRF1 complex

## Discussion

ChIP-seq/ChIP-chip techniques are powerful methods for identifying TF binding sites. However, these approaches currently are prone to a high false positive rate in predicting target genes [6, 7]. Therefore, we employed TIP [9] to remove binding peaks not located in predicted target genes and obtain better results than SpaMo [5] (Fig. 2a and b). Due to CST predicting TF complexes based on SpaMo, CST and SpaMo have similar curve treads in Fig. 2a. Other than TIP, many other methods exist for scoring target genes, such as TFAS [15] and ClosestGene [16], which can also be used to predict rankings. These methods all require binding peaks from a peak-calling algorithm [17–19]. Notably, the number of binding peaks is sensitive to the parameters of the peak-calling algorithm and thus can affect the accuracy and consistency of target gene prediction.

A previous study showed that USF2 and NFYA form TF complexes on the HoxB4 promoter in the K562 cell line [12], but this observation was not detected by CST. Our further scrutiny found that, among 9,428 narrow peaks from ENCODE K562 USF2 ChIP-seq data [20], there are no narrow peaks on the HoxB4 putative promoter (+/-3kbp around the TSS). We postulated that this is why CST was not able to detect it. To prevent such incidents from occurring, we suggest that the criteria for calling narrow peaks should be loosened. In CST, we used SpaMo to facilitate the prediction of TF complexes. SpaMo can predict whether two TFs belong to the same complex [5], but cannot confirm whether the interactions of the TF pair are direct or indirect. For example, the different motif spacings of the USF2-NFYA complex on different promoters (HoxB7, 21 bps; and HoxB4, 10 bps [12]) in K562 cells may arise from different interactions or conditions. USF2 and NFYA may interact indirectly when binding to HoxB7 but directly when binding to HoxB4, indicating that binding to HoxB7 may require more protein components than binding to HoxB4. This rationale may explain the observation in our qPCR experiments that the USF2-NFYA complex exhibited a higher binding affinity and enrichment on the HoxB7 promoter than on the HoxB4 promoter (Fig. 3a and b).

The accuracy of CST-predicted TF complexes from HeLa S3 NFYA ChIP-seq data can be confirmed by many studies (Table 3). Of the 7 CST-predicted TF complexes for NFYA in HeLa S3 cells, 5 have been previously reported: FOS [11], RFX5 [21], SREBP2 [22], TBP [23], and SP1 [2]. The experimental techniques involved in the above studies included immunoprecipitation, mammalian two-hybrid assays or luciferase assays for TF complex identification. Furthermore, CST-predicted TF complexes are supported by the published molecular structure data. Our results indicated that both IRF3-JUN and NFKB-IRF3 are TF complexes, consistent with a crystal structure in which ATF2/JUN, IRF3/IRF7 and NFKB form an enhanceosome on the interferon beta enhancer [24].

**Table 3:** Literature approval of the predicted TF complex formation from NFYA ChIP-seq data in HeLa S3 cells

Partner binding motif^a^	Predicted partner^b^	SpaMo *p*-value^c^	Reference^d^
E HeLa S3/FOS	FOS	5e-06	Fleming et al., [11]
E Sknsh/RFX5	RFX5	3.6e-05	Jabrane-Ferrat et al., [21]
E Hep G2/SREBP2	SREBP2	0.0015	Dooley et al., [22]
J NFIC	NFIC	0.0015	NA
J TBP	TBP	0.0076	Lee et al., [23]
E GM12878/TBLR1	SP1	0.014	Ravasi et al., [2]
E GM12878/CDP	SP1	0.015	Ravasi et al., [2]
E H1hesc/Rad21	Rad21	0.018	NA
J SP1	SP1	0.021	Ravasi et al., [2]
E K562/GTF2B	TBP	0.022	Lee et al., [23]
E Hep G2/MAZ	SP1	0.023	Ravasi et al., [2]

^a^The source of the partner binding motif. Summary names are used in the first column, in which “E HeLa S3/FOS” indicates the secondary motif from the ENCODE FOS ChIP-seq sample in the HeLa S3 cell line, and “J NFIC” indicates the motif from the JASPAR NFIC motif

^b^The list of the NFYA-partner TF complexes

^c^The p-value for the significant spacing of the binding motifs from SpaMo

^d^The external studies that support the existence of the TF complex

NA: reference is not available

In addition, we found that complex formation is dynamically changed in various conditions, even from the same cells. For example, the TF partners of MYC in K562 cells treated with interferon gamma are different from that with interferon alpha (Additional file 1: Table S4). Furthermore, even the same drug treatment, the different time points showed different complex formation. Notably, MYC appears to interact with the AP1 family in K562 cells exposed to interferon, independent of gamma or alpha subtype (Additional file 1: Table S4). The similar results were also observed in the TF partners of JUN in K562 cells (Additional file 1: Table S5).

The attraction of CST for predicting condition-specific TF complexes arises from its rich database containing vast vertebrate TF motifs. Thus, the results of CST are not hindered by cell lines that have little ChIP-seq data. There are thousands published ChIP-seq datasets, among which the MCF-7 cell line has a maximum of 40 distinct TF ChIP-seq datasets [25]. When Chen et al. [8] proposed an algorithm to identify TF complexes using paired ChIP-seq data, there were only 780 (*C*_2_^40^) potential TF complexes for scrutiny in MCF-7 cell lines. By contrast, there are 966 distinct vertebrate TF motifs in the TRANSFAC database (version 2013.2), resulting in 966 × 40 potential combinations for CST. Hence, CST is powerful and insightful, particularly for cell lines having few ChIP-seq datasets.

We are confident that CST will be helpful for detecting condition-specific TF complexes and their target genes because of its top-performing methods for target gene prediction (TIP [9]) and for partner TF prediction (MEME, FIMO and SpaMo [5, 26, 27]). Current bioinformatics approaches of TF target genes do not consider the fluidity of TF complexes [28]. Therefore, many important nuances in TF function and transcriptional regulation are missing. For example, USF2 could regulate iron transport and DNA catabolic processes when forming TF complexes with IRF1 and NFYA, respectively (Table 2). However, the top 10 GO terms of USF2 target genes identified by processing USF2 ChIP-seq data in the K562 cell line using the TIP algorithm (see Methods) are related to chromatin structure (Additional file 1: Table S6). If we only examine the GO results for USF2 targets, we may ignore important regulatory functions of USF2, including iron transport and DNA catabolic processes from the USF2-IRF1 and USF2-NFYA complexes, respectively.

## Conclusions

To the best of our knowledge, CST is the first pipeline that infers both condition-specific TF complexes and their target genes using human ChIP-seq datasets. Integrating the results of CST pipeline from 359 ChIP-seq ENCODE datasets, we constructed DBCST database. DBCST provides a searchable platform for TF complex and regulatory function discovery. DBCST is not only a database but also a web server and can perform CST pipeline from user’s own ChIP-seq experiment. User also can download CST package, which reports the list of primary TF targets and its binding sites when inputting wig file and narrow peak data, from DBCST download function. Using the result of CST package and then running MEME, FIMO and SpaMo, user can perform CST pipeline in their computer. We hope that DBCST will be a useful resource and provide insightful assistance for biologists studying transcriptional regulation going forward.

## Methods

### Data collection

Wiggle files and narrow peak data of 359 SYDH (Stanford/Yale/USC/Harvard) ENCODE ChIP-seq experiments were downloaded from the UCSC Genome Browser [29]. One hundred forty-six TF binding motifs were collected from the JASPAR CORE database [30]. To obtain a complete set of TF binding motifs across various conditions/cell types, we added 278 *de novo* motifs extracted from ENCODE ChIP-seq data using MEME [27] to our motif database. Genomic sequences and annotation files for RefSeq genes (both in hg19 version) were downloaded from the UCSC Genome Browser [20]. GO annotations were retrieved from the gene2go file (Dec 2012 version) on the NCBI Entrez Gene FTP site (ftp://ftp.ncbi.nlm.nih.gov/gene) [31].

### CST pipeline

The main steps of the CST pipeline are described below (Fig. 1).

#### Step 1. Identify target genes using TIP

Conventionally, TF target genes are identified by first selecting the binding peaks of the TF using a peak-calling algorithm (e.g., MACS [18]) and then by finding the genes with peaks in their putative promoters. However, this approach is known to produce many false positive target genes [6, 7]. In CST, the TF target genes are predicted using the Target Identification from Profiles (TIP) method [9] (Fig. 1a), which evaluates the confidence score of each putative target gene using a probabilistic model based on ChIP-chip or ChIP-seq data. TIP is one of the most accurate TF target gene prediction methods [16]. For all 359 ENCODE ChIP-seq samples, the selected TIP-derived target genes had to pass a confidence threshold of Q-value < 0.1.

#### Step 2. Identify binding peaks within target gene promoters

To examine TF binding motifs and their relative spacing, for each primary TF, the locations of binding peaks at the promoters of TIP-predicted target genes must first be identified. To accomplish this goal, we used the ENCODE narrow peak data to search for these peaks (Fig. 1b). Putative promoters were defined as the genomic regions +/- 3 kbp starting from the TSSs of the target genes. These regions are where the highest densities of accumulative TF binding peaks and histone modification signals both occur [32].

#### Step 3. Discover primary TF binding motifs using MEME

To discover primary TF binding motifs, we retrieved 120bps DNA sequences centred at the summits of the top 500 binding peaks (ranked by *p*-value) and used MEME (version 4.9.0_4 in the MEME suite) [27] with the “–mod zoops –maxw 10” options (Fig. 1c).

#### Step 4. Locate the primary TF binding motif using FIMO

A given binding peak may contain different motifs other than the primary binding motif for a primary TF. We employed FIMO (version 4.9.0_4 in the MEME suite with a *p*-value < 1e-4) [26] to select peaks containing the primary binding motif for each primary TF (Fig. 1d).

#### Step 5. Identify TF partners using SpaMo

We selected 300 bps DNA sequences centred on the primary binding motif after referencing a thermodynamic model of TF-TF interactions [33] in which 150 bps were the maximum distance for TF interactivity. These sequences were then used in SpaMo (version 4.9.0_4 in MEME suite) [5] to search for the presence of binding motifs of potential partner TFs (Fig. 1e).

SpaMo identified TF-TF pairs with enriched spacing between the primary and secondary motif. Given intervals centred on the primary TF binding sites from the ChIP-seq data to identify the significant spacing between motif pairs, SpaMo assumes that the number of observed spacings between the primary TF and the secondary TF motifs follows a binomial distribution. Our pipeline for the secondary binding motifs included the *de novo* motifs identified in step 3 (278 in total; derived from the ENCODE data) and 146 additional motifs from the JASPAR CORE motif database [30]. The primary-secondary TF pair is reported when the spacing between the primary and secondary motif was significant (SpaMo *p*-value < 0.05 and E-value < 10).

#### Step 6. Report the target genes with GO enrichment analysis for each TF complex

GO enrichment analysis was performed for predicted target genes (Fig. 1f) using Fisher’s exact test scores based on the hypergeometric distribution for each GO term. CST provides two sets of target genes: high-confidence and low-confidence. A high-confidence target gene is called if the following standards are fully met: (i) it is the target gene for a primary TF; (ii) it has a primary and a secondary motif on its promoter, and its motif spacing is ≤ 150 bases [33]; and (iii) the spacing of the motif pair is significant (SpaMo *p*-value < 0.05 and E-value < 10). By contrast, a low-confidence target gene is called if standard (iii) is not met. The latter is used to describe TFs with variable spacing because of their binding on wrapped DNA strings or nucleosomes.

### Validation

To validate our results, we employed a three-step approach: (1) comparison of predicted TF complexes against an external and empirically derived TF complex database; (2) spot validation of the target genes using ChIP-qPCR and RT-PCR; and (3) GO enrichment analysis of the target genes.

#### Step 1. Comparison of predicted TF complexes against external TF complex databases

Two procedures were used in this step. First, we compared our list of TF complexes to a TF-TF interaction database experimentally derived and collected by Ravasi [2]. To calculate their enrichment ratios against this database, we rank ordered our predicted TF complexes based on their *p*-values and then examined the TF complexes in groups (TF complexes with the lowest 10% of *p*-values followed by TF complexes with the lowest 20% of *p*-values up to 100% of TF complexes). Similar steps were conducted on TF complexes predicted using SpaMo as a reference. Next, enrichment ratios for CST and SpaMo were determined relative to a randomly generated list of TF complexes in CST or SpaMo based on the following formula: the observed number (CST or SpaMo TF complexes in the Ravasi database) divided by the expected number (randomly generated TF complexes in the Ravasi database).

Second, we used another external TF repository of transcriptional regulatory modules (TRMs) [10] for further comparisons. Briefly, we conducted hypergeometric distribution Fisher’s exact tests on the degrees of overlap between CST and TRMs to check for consistency.

#### Step 2. Validation of CST-predicted target genes using ChIP-qPCR and RT-PCR

Chromatin immunoprecipitation quantitative PCR (ChIP-qPCR). Four ChIP-qPCR experiments for the NFYA and USF2 TFs in the K562 and HeLa cell lines were performed using a selection of the NFYA-USF2-predicted TF complex target genes. HoxB4, a literature-derived external positive control from Zhu et al., was one of the qPCR amplification targets [12]. ChIP was performed using a ChIP kit (Millipore, Billerica, MA, USA) according to the manufacturer’s instructions and described in the Additional file 1 Supplementary Methods. The selected qPCR amplification targets were HoxB7, GLYR1 and EIF4E (which were derived from the CST-predicted target gene list of the NFYA-USF2-predicted TF complex) and HoxB4 (which was derived from external work confirming NFYA-USF2 regulation and used as a positive control [12]). The forward and reverse primers used for the ChIP-qPCR are listed in Additional file 1: Table S3. The locations of these primers are illustrated in Additional file 1: Fig. S5.

Real-time reverse transcription-polymerase chain reaction (RT-PCR) and Western blotting. USF2-specific siRNA (SI02780785) and scrambled control were purchased from Qiagen (Massachusetts, USA) and employed to silence the USF2 expression in HeLa cells. Subsequently, total RNAs were purified by Trizol reagent (Invitrogen) and then subjected to SYBR Green RT-PCR using an ABI Prism 7300 sequence detection system (Applied Biosystems, Philadelphia, PA, USA), as described previously [34]. The primers used for amplification are listed in Additional file 1: Table S3. The expression of the mRNA normalized to that of the internal control (TATA box-binding protein, TBP) was defined as -ΔCT = -(CT_Target_ - CT_TBP_), whereas the relative expression of the target gene was calculated using the 2^–ΔCT^ method. The detailed procedures of immunoblotting were performed as described previously [35]. The antibodies included anti-USF2 (Abcam, Burlingame, CA, USA) and anti-β-tubulin (Millipore, Bedford, MA, USA), primary antibody as well as horseradish peroxidase-conjugated secondary antibody (Santa Cruz Biotechnology Inc.). The β-tubulin acted as an internal control.

#### Step 3. Gene ontology enrichment analysis of the target genes of selected TF complexes

To validate that CST captured the phenomenon mentioned in Mullen, A. C. *et al* [1], we performed GO enrichment analysis for target genes of a primary TF with a different partner TF.

## Additional file

Additional file 1: Supplementary Instruction for Browsing Web Interface and Supplementary Methods. **Figure S1.** The likelihood of TF complexes near a transcriptionally active gene. **Figure S2.** High-confidence and low-confidence target genes. **Figure S3.** SpaMo-predicted TF complexes from GATA2 and TAL1 ChIP-seq experiments in K562 cells. **Figure S4.** ChIP-PCR and slab gel electrophoresis against CST NFYA-USF2-predicted target genes. **Figure S5.** The schema of ChIP-PCR primers. **Figure S6.** DBCST’s partners view. **Figure S7.** DBCST’s network view. **Figure S8.** DBCST’s target genes view. **Figure S9.** DBCST’s upload functionality. **Table S1.** Statistics for the TF and ChIP-seq datasets used in the construction of DBCST for each cell line. **Table S2.** The intersection of TF-TF interactions predicted by TRMs and CST. **Table S3.** A list of PCR primers. **Table S4.** Dynamic TF complexes and binding motifs from K562 MYC ChIP-seq data in various conditions. **Table S5.** Dynamic TF complexes and binding motifs from K562 JUN ChIP-seq data in various conditions. **Table S6.** A partial list of the GO analysis results of target genes predicted by USF2 ChIP-seq data in the K562 cell line. (PDF 1190 kb). (DOC 2001 kb)

## Abbreviations

ChIP-seq: chromatin immunoprecipitation sequencing
CST: Condition-Specific Target
DBCST: Database of Condition-Specific Targets
qPCR: Quantitative polymerase chain reaction
RT-PCR: Reverse transcription-PCR.
TF: Transcription factor
TRM: Transcriptional regulatory module
TSS: Transcription start site
