# Supplementary material for: Inferring condition-specific targets of human TF-TF complexes using ChIP-seq data
Source: BMC Genomics. 2017 Jan 10;18:61. doi: 10.1186/s12864-016-3450-3 (PMC5223348; doi:10.1186/s12864-016-3450-3)
Supplement: Additional file 1: — Supplementary Instruction for Browsing Web Interface and Supplementary Methods. Figure S1. The likelihood of TF complexes near a transcriptionally active gene. Figure S2. High-confidence and low-confidence target genes. Figure S3. SpaMo-predicted TF complexes from GATA2 and TAL1 ChIP-seq experiments in K562 cells. Figure S4. ChIP-PCR and slab gel electrophoresis against CST NFYA-USF2-predicted target genes. Figure S5. The schema of ChIP-PCR primers. Figure S6. DBCST’s partners view. Figure S7. DBCST’s network view. Figure S8. DBCST’s target genes view. Figure S9. DBCST’s upload functionality. Table S1. Statistics for the TF and ChIP-seq datasets used in the construction of DBCST for each cell line. Table S2. The intersection of TF-TF interactions predicted by TRMs and CST. Table S3. A list of PCR primers. Table S4. Dynamic TF complexes and binding motifs from K562 MYC ChIP-seq data in various conditions. Table S5. Dynamic TF complexes and binding motifs from K562 JUN ChIP-seq data in various conditions. Table S6. A partial list of the GO analysis results of target genes predicted by USF2 ChIP-seq data in the K562 cell line. (PDF 1190 kb). (DOC 2001 kb) [file 12864_2016_3450_MOESM1_ESM.doc]

**Supplementary Information**

**Inferring condition-specific targets of human TF-TF complexes
using ChIP-seq data**

Chia-Chun Yang, Min-Hsuan Chen, Sheng-Yi Lin, Erik H. Andrews, Chao Cheng, Chun-Chi Liu, Jeremy J.W. Chen

**Correspondence:** Jeremy J.W. Chen, Institute of Biomedical Sciences, National Chung-Hsing University, No. 250, Kuo-Kuang Rd., Taichung 40227, Taiwan. Phone: 886-4-22840896 ext. 125; Fax: 886-4-22853469; Email: jwchen@dragon.nchu.edu.tw

**1. Supplementary Instruction for Browsing Web Interface:**

DBCST has two major functions: (i) searching TF complexes and target genes currently residing in the database and (ii) uploading new ChIP-seq data to investigate TF complexes. The following is a brief introduction for browsing the DBCST webpage. A free, detailed tutorial is available on the DBCST webpage (<http://syslab3.nchu.edu.tw/DBCST/>).

**1.1. Searching TF complexes and target genes**

There are three views in the search function as listed below.

***1.1.1. TF partners view:*** Users can select a TF with certain cell conditions to search associated TFs, and the server will provide a table of experiment names, primary motif logos, associated TFs, SpaMo-derived motif spacing p-values and high-confidence target genes. The experiment name consists of the cell line, primary TF, experimental laboratory and treatment conditions. For example, K562_IRF1_Sydh_IFNa6h indicates that the experiment used the IRF1 antibody on K562 cells with 6 hours interferon alpha treatment from the SYDH (Stanford/Yale/USC/Harvard) group. In the results table, when users click the TF partner name, the server provides detailed information regarding the primary motif, secondary motifs, motif spacing histograms, target genes and GO enrichment results. For example, when selecting the USF2 ChIP-seq sample in HeLa S3 cells, the server lists 11 TF partners sorted by SpaMo-derived motif-spacing p-values. From this result, USF2 is shown to interact with NFYA to form the USF2-NFYA complex and targets 14 high-confidence target genes (e.g., APEX1, CBR1, and DNAJB9) (Fig. S6).

***1.1.2. Network view:*** Because TF partners can determine the target genes of TF complexes, the visualization of dynamic target genes of TF complexes facilitates the study of condition-specific regulatory networks. Given a TF and a cell condition, DBCST generates a list of condition-specific significant TF partners and constructs the regulatory network of TF complexes and high-confidence target genes (Fig. S7).

***1.1.3. Target genes view:*** Users can input a gene symbol, and the server will generate a table of TF pairs that may target the given gene under certain cell conditions. In this view, the server only reports TF complexes for which the gene symbol is a high-confidence target gene. In the results table, when users click on the TF partner name, the server will link to its entry and provide detailed information on its primary motif, secondary motifs, motif spacing histogram, target genes and GO enrichment results (Fig. S8).

**1.2. Upload ChIP-seq data**

When experimental biologists generate phenotype-specific ChIP-seq data, it is often a challenge to process the data and then generate a biological hypothesis. To facilitate this analysis, users can upload the aligned reads and binding peak files from their ChIP-seq data to perform TF complex discovery and target gene identification. The server supports the Wiggle, BigWig and bedGraph track formats for the aligned read data. Binding peak data can be uploaded as a narrow peak file in either the UCSC or MACS14 format (Fig. S9). After selecting the parameters (species, Q value threshold of TIP and secondary motif database), the server takes approximately 18 minutes to perform the TF complex analysis and then outputs the TF complexes, target genes and GO enrichment results.

**2. Supplementary Methods:**

- 1. **The Correlation between expression and TF binding**

To demonstrate the correlation between expression and binding, we used K562 NFE2 ChIP-seq data and gene expression data after silencing NFE2 using siRNA[^1^](#_ENREF_1). For identifying the ranks of TF binding affinity, we use TIP algorithm. TIP is an algorithm to identify the rank of TF targets by calculating TF binding affinity from ChIP-seq data. After calculating the correlation of binding affinity (from TIP) and gene expression (from absolute of log(treat/control)), we obtained correlation coefficient 0.25 (*P* = 2.2 × 10^-16^), suggesting significantly high correlation between gene expressions and TF bindings.

- 1. **Chromatin immunoprecipitation**

For each experiment, 2 × 10^7^ K562 or HeLa cells were cross-linked with 1% formaldehyde. The nuclei were collected and sonicated to generate an average DNA fragment size of 200-1000 bp. The chromatin solution was incubated with protein A/G magnetic beads and an NFYA antibody from Santa Cruz Biotechnology (Santa Cruz, CA), a USF2 antibody (Santa Cruz, CA) or a nonspecific IgG antibody (Millipore) for 16 h at 4°C, and 1% chromatin solution was used as the input. The immune complexes were washed with low salt, high salt, LiCl and TE buffer followed by elution. The eluted solutions and input solution were incubated for 2 h with proteinase K at 65°C to reverse the cross-links and then purified using spin columns. For target gene amplification assessment, equal amounts of the purified IP-DNA and input-DNA were used to perform regular and quantitative PCR with the comparative C_T_ method. To determine the fold enrichment of target DNA in the NFYA-IP and USF2-IP samples relative to the target DNA in IgG-IP controls, ΔC_T_ was calculated by subtracting the C_T_ of the IgG-IP from the C_T_ of the NFYA-IP or USF2-IP. The amount of target DNA enrichment is calculated by the following formula: 2^-ΔCT^. The PCR conditions for the immunoprecipitated genomic DNA were as follows: 94°C for 3 minutes, denaturing at 94°C for 20 s, annealing at 59°C for 30 s, and extension at 72°C for 30 s, for 32-35 cycles.

**3. Supplementary reference:**

1 Gerstein, M. *B. et a*l. Architecture of the human regulatory network derived from ENCODE data*. Natu*r**e 4**89, 91-100, doi:Doi 10.1038/Nature11245 (2012).

**4. Supplementary Tables:**

**Supplementary Table S1. Statistics for the TF and ChIP-seq datasets used in the construction of DBCST for each cell line.**

|  | **Cell line** | **No. of distinct TFs^*^** | **No. of datasets^#^** |
| --- | --- | --- | --- |
| 1 | K562 | 58 | 77 |
| 2 | HeLa S3 | 43 | 43 |
| 3 | GM12878 | 40 | 40 |
| 4 | HepG2 | 28 | 30 |
| 5 | H1-hESC | 19 | 19 |
| 6 | MCF10A-Er-Src | 6 | 13 |
| 7 | A549 | 6 | 6 |
| 8 | IMR-90 | 6 | 6 |
| 9 | SK-N-SH | 6 | 6 |
| 10 | HEK293 | 4 | 4 |
| 11 | HUVEC | 4 | 4 |
| 12 | MCF-7 | 3 | 3 |
| 13 | GM18505 | 2 | 2 |
| 14 | GM18951 | 2 | 2 |
| 15 | GM19193 | 2 | 2 |
| 16 | NB4 | 2 | 2 |
| 17 | PBDE | 2 | 2 |
| 18 | SH-SY5Y | 2 | 2 |
| 19 | GM10847 | 1 | 1 |
| 20 | GM12891 | 1 | 1 |
| 21 | GM12892 | 1 | 1 |
| 22 | GM15510 | 1 | 1 |
| 23 | GM18526 | 1 | 1 |
| 24 | GM19099 | 1 | 1 |
| 25 | HCT-116 | 1 | 1 |
| 26 | NT2-D1 | 1 | 1 |
| 27 | PANC-1 | 1 | 1 |
| 28 | PBDEFetal | 1 | 1 |
| 29 | U2OS | 1 | 1 |

*The number of TFs in the ChIP-seq datasets for the indicated cell line.

^#^The number of ChIP-seq datasets available for the cell line. For example, ENCODE contains two ChIP-seq datasets specific for CTCF using SC-15914 and SC-5916 antibodies in the K562 cell line. Therefore, the number of distinct TFs is 1, and the number of datasets is 2.

**Supplementary Table S2. The intersection of TF-TF interactions predicted by TRMs and CST.**

| **TF-TF complex** | **SpaMo p-value** | **Spacing of motif pairs*** | **Original TRM^#^** |
| --- | --- | --- | --- |
| TAL1-ELK1 | 6.7e-04 | 7 bp | TAL1 TRM in HSCs |
| TAL1-STAT1 | 2.1e-03 | 85 bp | TAL1 TRM in HSCs |
| TAL1-GATA1 | 0.014 | 23 bp | TAL1 TRM in HSCs |
| GATA2-TAL1 | 0.019 | 23 bp | GATA2 TRM in HSCs |

*The predicted spacing of binding motifs of the primary TF and partner TF.

^#^The TRM containing the indicated TF-TF complex formation. “TAL1 TRM in HSCs” indicates the TRM from Tal1 ChIP-seq data in HSCs.

**Supplementary Table S3. A list of PCR primers.**

| **Genes** | **forward** | **reverse** |
| --- | --- | --- |
| **Primer of ChIP-qPCR** | | |
| HoxB7-1 | 5’-AACTCATAATTTGGCCGGATG-3’ | 5’-CACATTACCGGGAGCCGTGG-3’ |
| HoxB7-2 | 5’-AGGACCCCCTTTTTCCTCT-3’ | 5’-CTCGGCTTTCCCATTCAT-3’ |
| GLYR1 | 5’-GCCGCTACTCTGACGTCACCG-3’ | 5’-TTGTCACGAGCCGAAGGGAG-3’ |
| EIF4E | 5’-ATCTGATCGCACAACCGCT-3’ | 5’-TTCCCAGAAGCCTCTCGTTAC-3’ |
| HoxB4 | 5’-CGAAAGCCCTCCTACTTACTGTC-3’ | 5’-GGAAATAAACCTCTTTGGCTGG-3’ |
| **Primer of real time RT-PCR** | | |
| USF2 | 5’-TGGATCGTCCAGCTTTCGAA-3’ | 5’-AAGGTCTCCTGCATGCGCT-3’ |
| HoxB7 | 5’-GCCGAGAGTAACTTCCGGATCT-3’ | 5’-CGCGTCAGGTAGCGATTGTAGT-3’ |
| EIF4E | 5’-ACGGAATCTAATCAGGAGGTTGC-3’ | 5’-AACTTGGAGATCAGCCGCAG-3’ |
| GLYR1 | 5’-GTCACACAGTGACTGTCTGGAACC-3’ | 5’-ACGCAGGCGAAAGTGATGTC-3’ |

**Supplementary Table S4. Dynamic TF complexes and binding motifs from K562 MYC ChIP-seq data in various conditions.**

| Condition | MYC ChIP-seq binding motif in K562 cell lines | Predicted TF partners |
| --- | --- | --- |
| Standard | 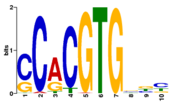 | ZEB1 |
| Treatment with interferon gamma for 30 minutes | 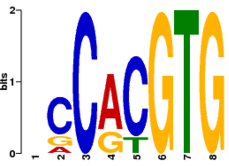 | IRF1, FOS, SPI1 |
| Treatment with interferon gamma for 6 hours | 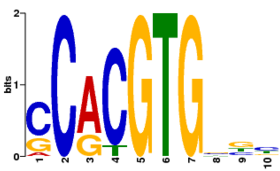 | JUND, TCF7 |
| Treatment with interferon alpha for 30 minutes | 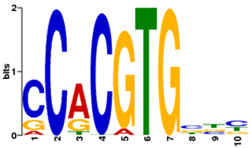 | MYC |
| Treatment with interferon alpha for 6 hours | 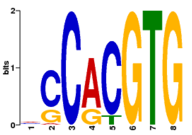 | JUN, MXI1, ZNF143 |

**Supplementary Table S5. Dynamic TF complexes and binding motifs from K562 JUN ChIP-seq data in various** conditions.

| Condition | JUN ChIP-seq binding motif in K562 cell lines | Predicted TF partners |
| --- | --- | --- |
| Standard | 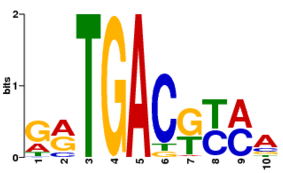 | No predicted TF partners |
| Treatment with interferon gamma for 30 minutes | 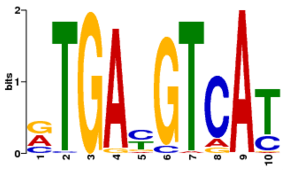 | TEAD1, MXI1, MYC, IKZF1 |
| Treatment with interferon gamma for 6 hours | 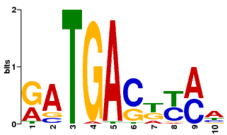 | TBP, MIZF |
| Treatment with interferon alpha for 30 minutes | 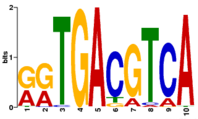 | RFX5 and TR4 |
| Treatment with interferon alpha for 6 hours | 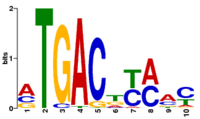 | No predicted TF partners |

**Supplementary Table S6. A partial list of the GO analysis results of target genes predicted by USF2 ChIP-seq data in the K562 cell line.**

| **Rank** | **Enrichment GO term** | **p-value** |
| --- | --- | --- |
| **1** | GO:0000786 nucleosome | 1.55E-14 |
| **2** | GO:0006334 nucleosome assembly | 2.95E-12 |
| **3** | GO:0034728 nucleosome organization | 8.20E-12 |
| **4** | GO:0065004 protein-DNA complex assembly | 1.82E-11 |
| **5** | GO:0032993 protein-DNA complex | 3.26E-11 |
| **6** | GO:0071824 protein-DNA complex subunit organization | 3.29E-11 |
| **7** | GO:0044437 vacuolar part | 1.11E-10 |
| **8** | GO:0046907 intracellular transport | 4.29E-10 |
| **9** | GO:0044427 chromosomal part | 4.84E-10 |
| **10** | GO:0005773 vacuole | 7.69E-10 |

Top 10 GO enrichment results for USF2 targets. The targets of USF2 were identified by analysing USF2 ChIP-seq data in the K562 cell line using the TIP algorithm with Q-values < 0.1 being declared as significant.

**5. Supplementary Figures:**


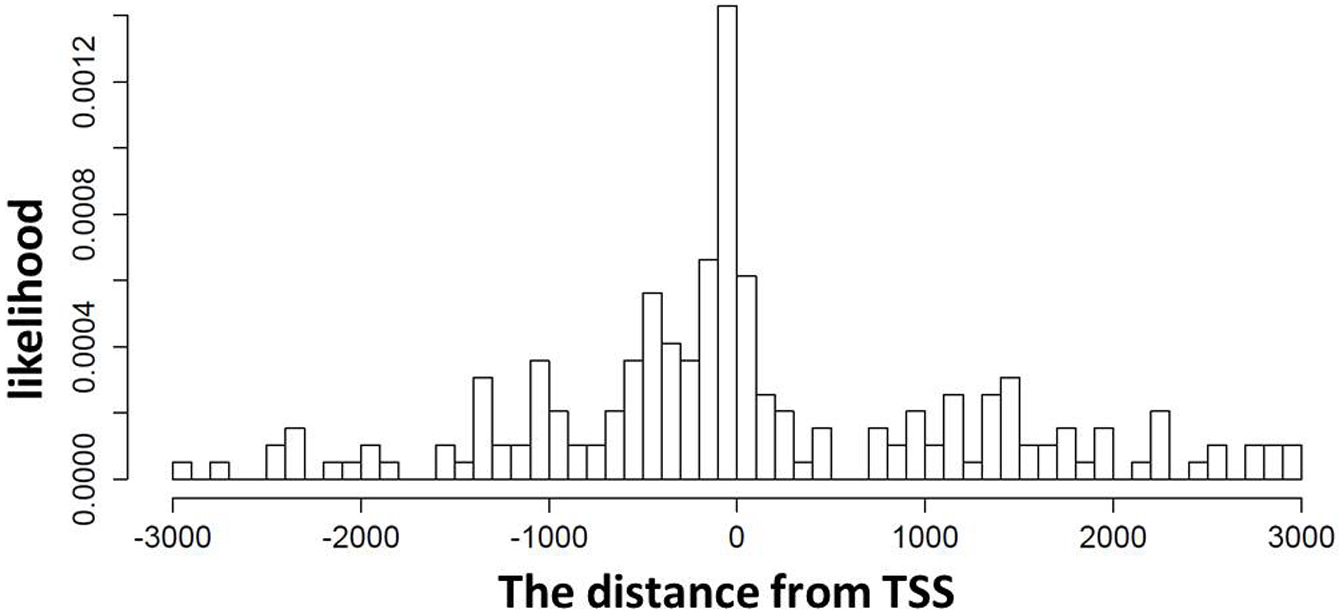


**Supplementary Figure S1**. **The likelihood of TF complexes near a transcriptionally active gene.** To demonstrate the likelihood of a TF complex lying near transcriptionally active genes, we performed the following analysis: (1) integrate six K562 ChIP-seq and gene expression data after silencing GATA1, GATA2, JUND, NFE2, RAD21 and CTCF using siRNA; (2) identify fold changes of gene expression >=1.5 as the active genes, and obtain 131 transcriptionally active targets of TF complexes having 197 binding sites; (3) bin the 197 distances of complex binding sites from TSS into 100-bp bins, and estimate the likelihood of a TF complex near a transcriptionally active gene. The result suggested that TF complexes are most likely located -1 kbp to 0.5 kbp around TSS of transcriptionally active genes.


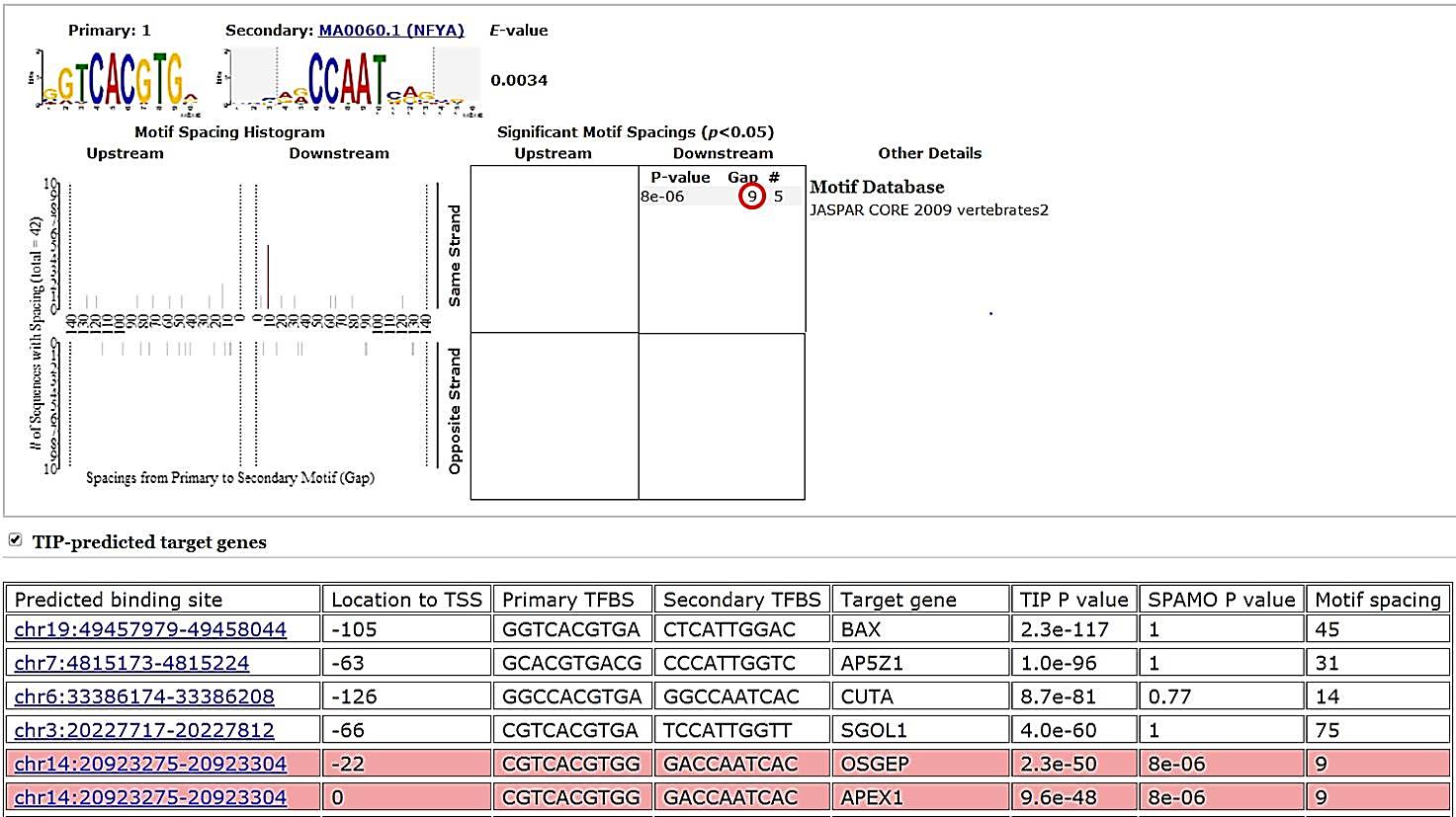


**Supplementary Figure S2. High-confidence and low-confidence target genes.** The results of TF complex prediction using the K562 USF2 ChIP-seq data. SpaMo reports that the USF2 and NFYA motif spacing was most significant at 9 bp. The high-confidence target genes are highlighted in red (motif spacing is 9 bp).


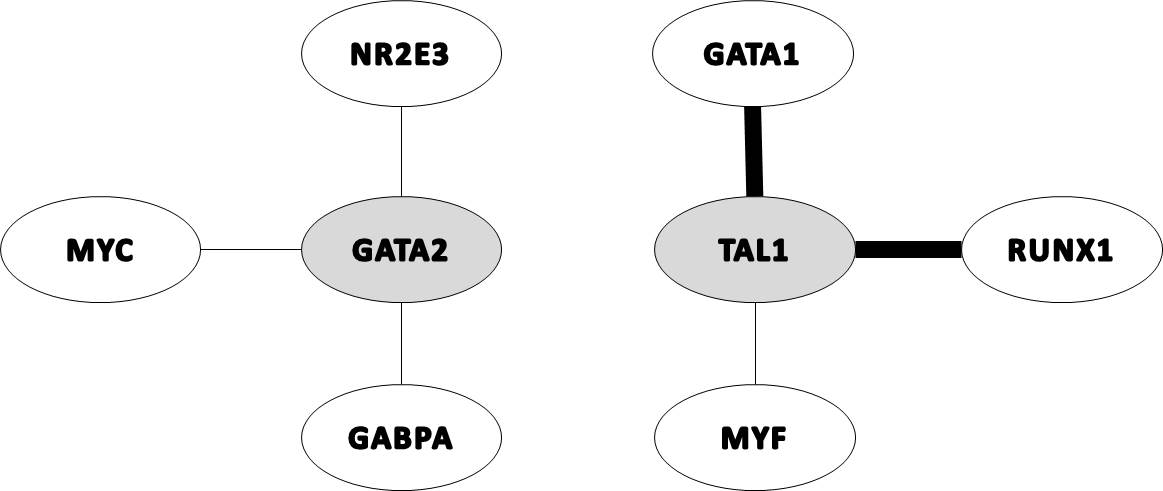


**Supplementary Figure S3. SpaMo-predicted TF complexes from GATA2 and TAL1 ChIP-seq experiments in K562 cells.** There are 6 TF-TF interactions predicted by SpaMo using GATA2 and TAL1 ChIP-seq experiments in K562 cells and two significant TF complexes between TRMs and SpaMo are indicated with bold edges (*P*=0.02; Fisher’s exact test).


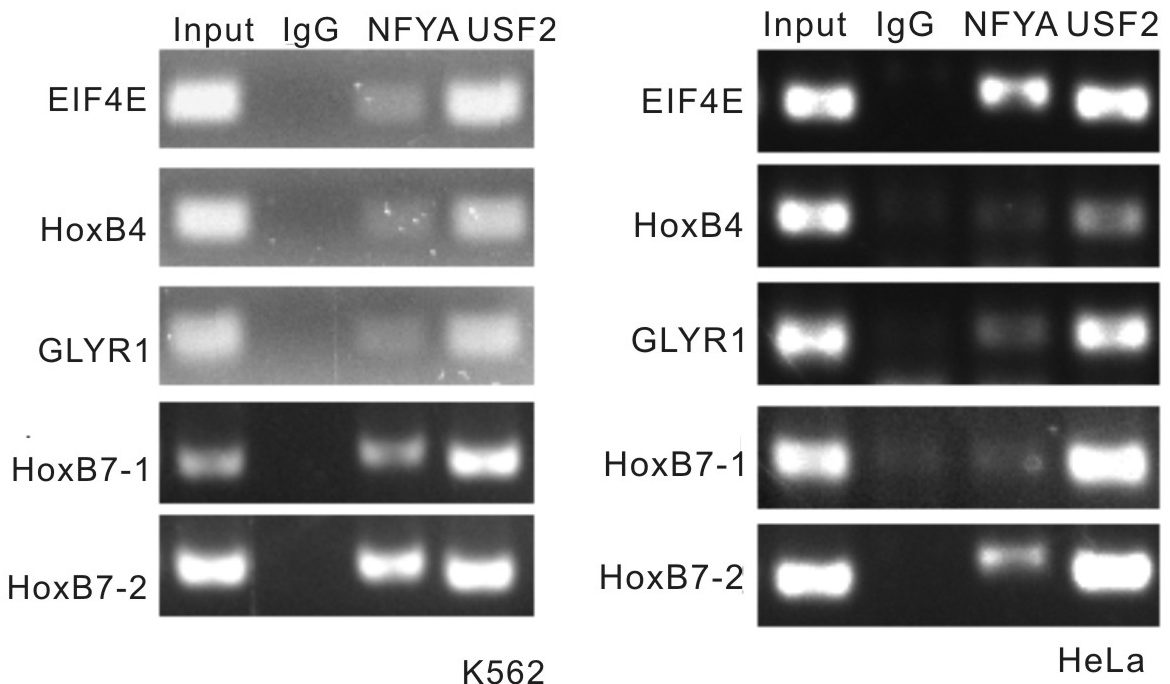


**Supplementary Figure S4. ChIP-PCR and slab gel electrophoresis against CST NFYA-USF2-predicted target genes.** PCR amplifications from input, IgG-IP, NFYA-IP and USF2-IP DNA are shown for each gene promoter, as noted. IgG-IP DNA was used as a negative control, whereas input DNA was a positive control.


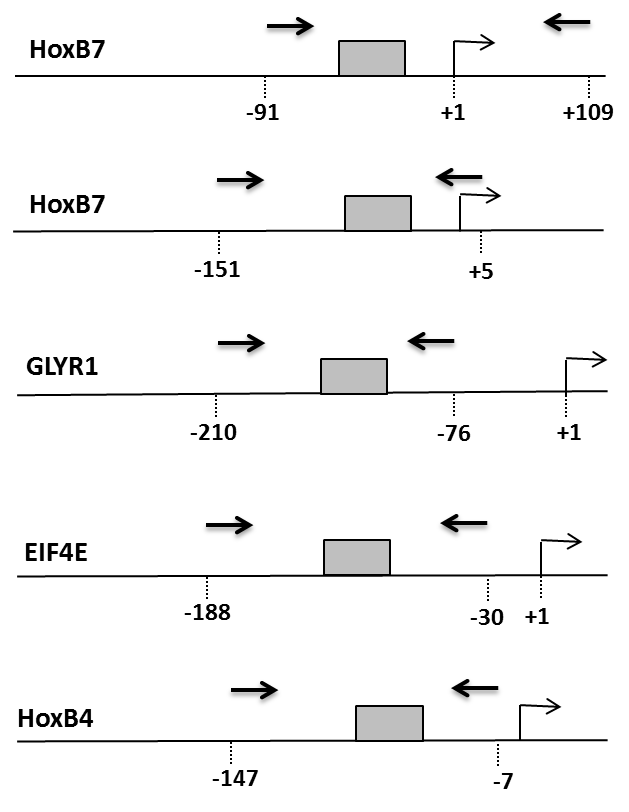


**Supplementary Figure S5**. **The schema of ChIP-PCR primers.** Left arrow indicates the forward primer; right arrow indicated the reverse primer. Grey rectangles represent the predicted binding sites of USF2-NFYA complex.


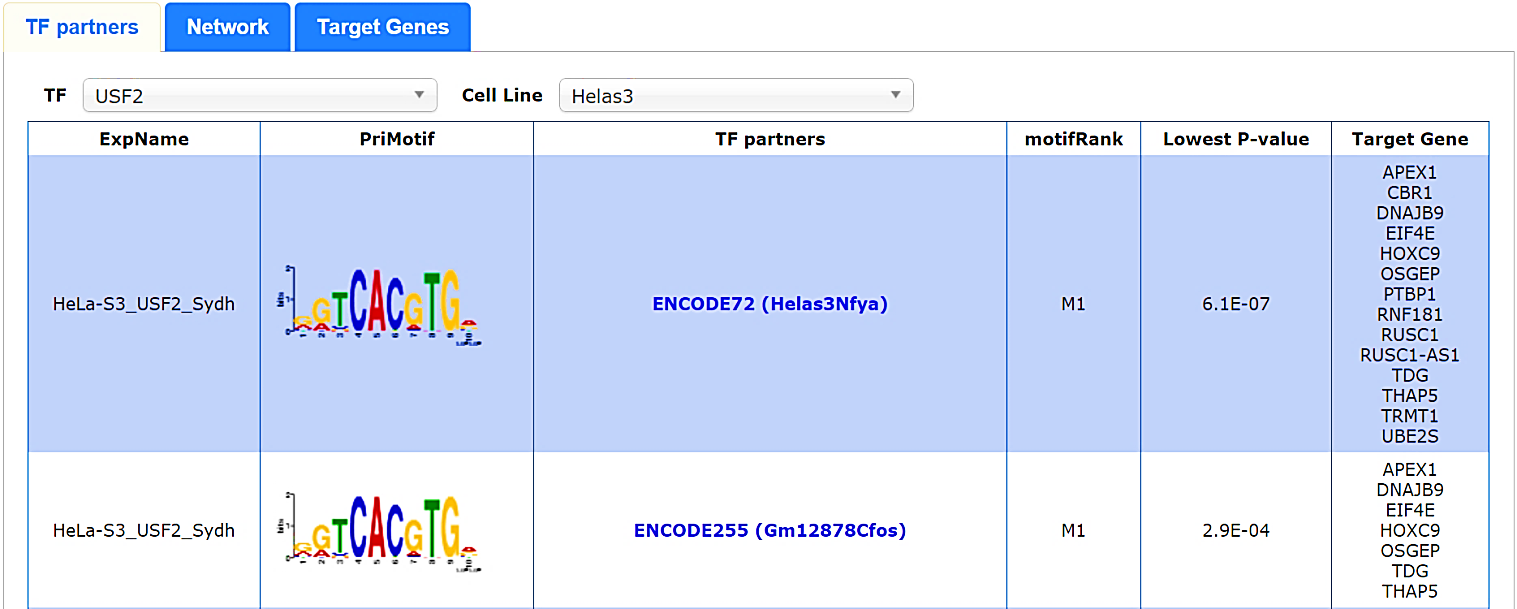


**Supplementary Figure S6. DBCST’s partners view.** Users can choose a TF and cell type to search for associated TF partners, and the server will provide a table of experiment names for ChIP-seq data, logos of primary motifs, associated TF partners, spacing p-values and high confidence target genes. When users click the TF partner name, the server will provide detailed information on the primary motif, secondary motifs, motif spacing histogram, target genes and GO enrichment results. For example, choosing the USF2 ChIP-seq sample in HeLa S3 cells, the server lists 11 TF partners sorted by SpaMo calculated p-values for their motif spacings. Based on these results, USF2 most likely interacts with NFYA to form the USF2-NFYA complex, which has 14 high-confidence target genes, including APEX1, CBR1, and DNAJB9.


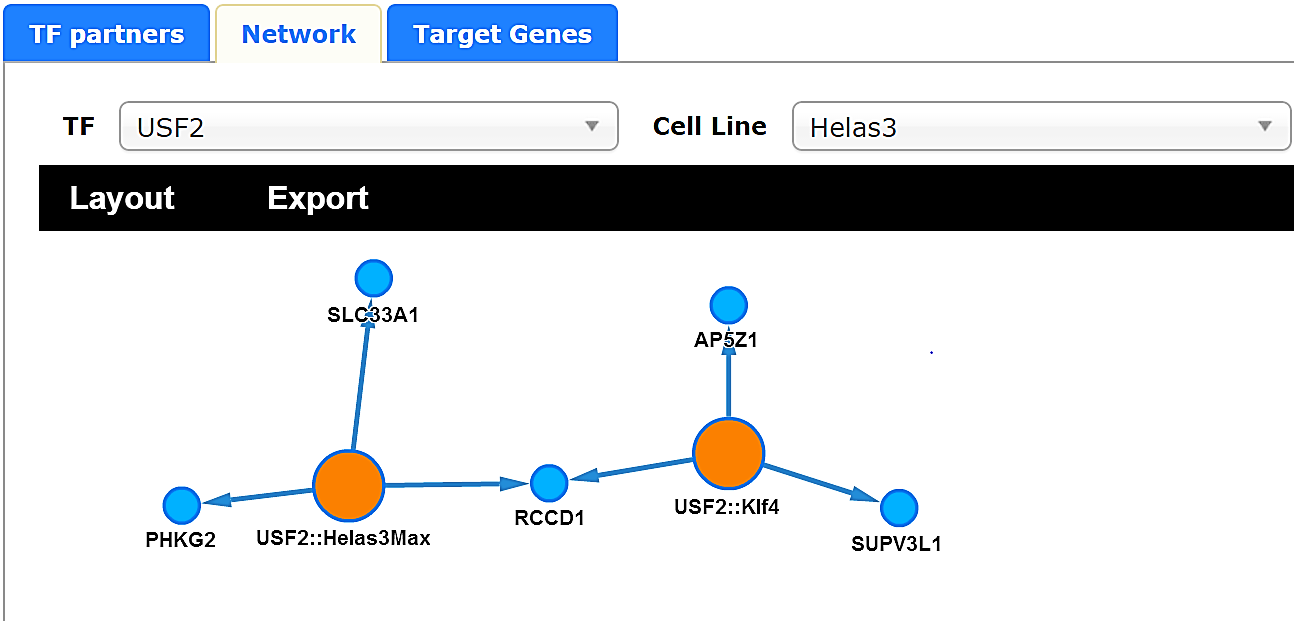


**Supplementary Figure S7. DBCST’s network view.** Given a TF and cell condition in Network view, DBCST will generate a list of condition-specific significant TF partners (p-value < 0.05 and E-value < 10 of SpaMo) and draw the regulatory network of TF complexes (orange circles) and high-confidence target genes (blue circles). For example, USF2::Max and USF2::KLF4 are predicted TF complexes in HeLa S3 cells, and both of the TF complexes contain the high-confidence target gene *RCCD1*. By clicking on the orange circle (a TF complex), the system will provide detailed information regarding that complex, including the ChIP-seq experiment name and its associated p-value.


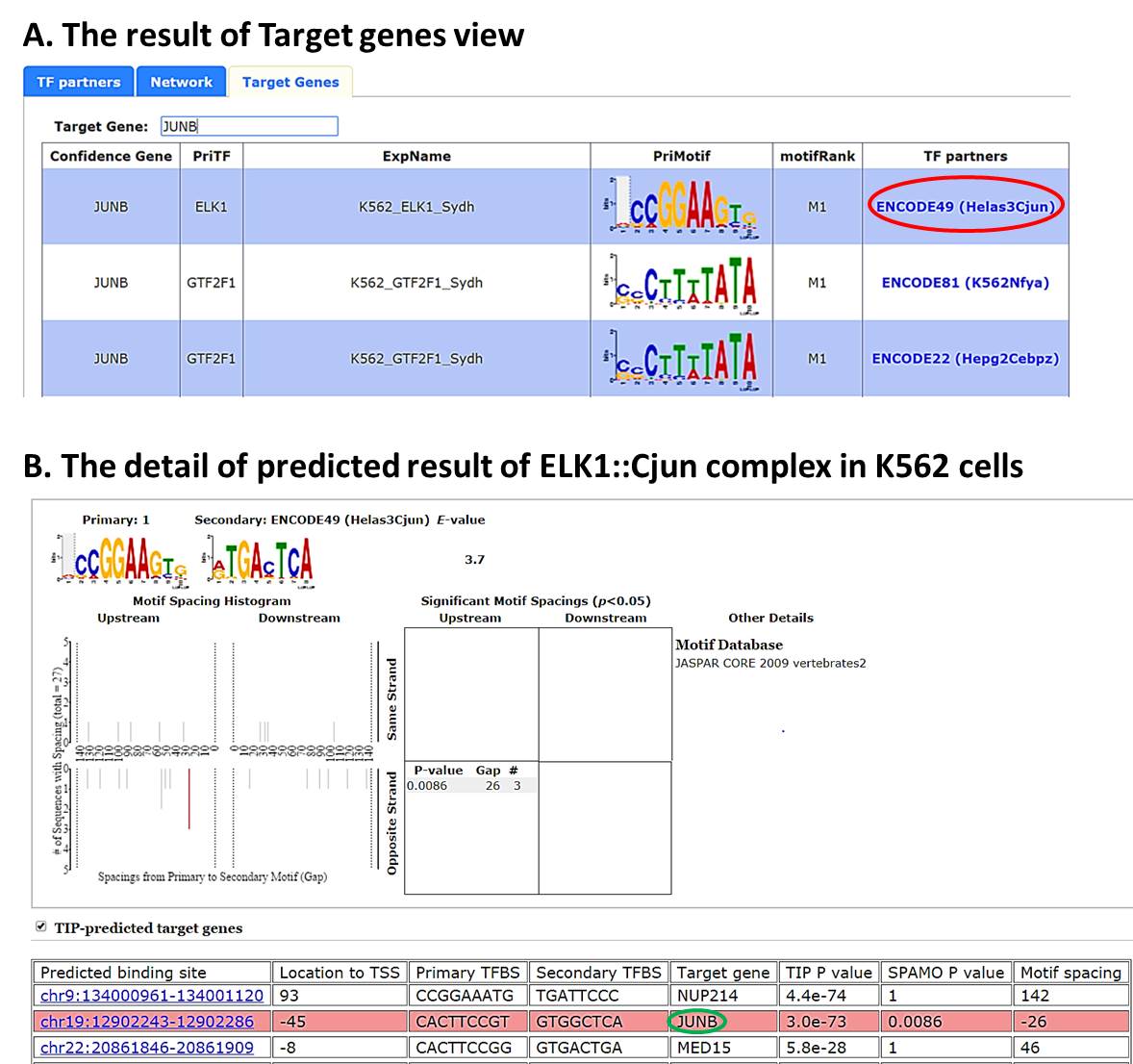


**Supplementary Figure S8. DBCST’s target genes view.** (A) Given a gene symbol, the server will generate a table of TF pairs that may target the given gene under certain cell conditions. This list only includes TF complexes for which the given gene is a high-confidence target. For example, the first row of the table indicates that ELK1 and CJUN are predicted to complex based on the ENCODE Sydh K562 ELK1 ChIP-seq data, and JUNB is the predicted high-confidence target gene of the complex. After clicking on the TF partner name in the first row (red circle, A), the server will provide (B) detailed information on the primary motif, secondary motifs, motif spacing histogram, target genes and GO enrichment results.


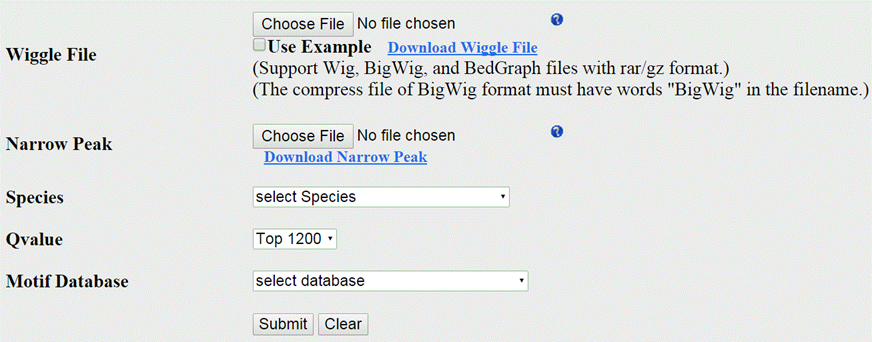


**Supplementary Figure S9. DBCST’s upload functionality.** DBCST also supports data upload for users to analyse their own data. Wiggle files in .rar or .gz format and narrow peak files are required for data processing. After uploading a Wiggle file and narrow peak file, users must select the parameters of species, Q value for TIP and Motif Database for SpaMo secondary motifs.
